# Enhancing vascular surgery outcomes through geriatric co-management: a study on the impact of the POPS team

**DOI:** 10.1308/rcsann.2024.0084

**Published:** 2024-11-12

**Authors:** MEA Bakheet, M Hughes, N Darwish, S Chen, A Egun, M Banihani

**Affiliations:** Lancashire Teaching Hospitals NHS Foundation Trust, UK

**Keywords:** Peripheral arterial disease, Length of stay, Critical limb ischemia, Geriatric co-management

## Abstract

**Introduction:**

Peripheral arterial disease (PAD) involves atherosclerotic stenosis and occlusion of lower leg arteries, leading to significant disability, high cardiovascular and cerebrovascular morbidity and mortality. Critical limb ischemia (CLI) is the most severe form of PAD. With the UK's aging population set to increase, the prevalence of PAD and the burden on vascular teams are expected to rise. This study evaluates the impact of regular input from the Proactive Care of Older People Undergoing Surgery (POPS) team on vascular surgery outcomes.

**Methods:**

This prospective cohort study examined the impact of Care of the Elderly (CoE) input on predefined parameters, focussing primarily on the length of stay (LoS) over 12 months. Data included baseline demographics, comorbidities, frailty scores (assessed using the Rockwood frailty score), LoS and referrals to medical specialties. A retrospective pilot study of 50 consecutive patients indicated a need for CoE input, showing higher local LoS compared with the national average.

**Results:**

Patients in both pilot and project groups were matched for comorbidities, frailty scores and interventions. Despite higher mean age and a greater proportion of patients aged 75+ years in the project group, the study aimed to reduce LoS. Post-quality improvement project implementation, LoS beyond fit-for-discharge decreased from 11.7 days to 9 days in 6 months and to 6 days after 12 months. Referrals to medical specialties decreased from 77% to 40%, and new diagnoses on discharge increased from 28% to 37%.

**Conclusions:**

CoE team input in vascular surgery patient care significantly improved outcomes, reducing LoS and medical specialty referrals, demonstrating cost-effectiveness and suggesting a feasible multidisciplinary approach for other regions.

## Introduction

Peripheral arterial disease (PAD) involves atherosclerotic stenosis and/or occlusion of lower leg arteries, leading to significant disability.^1^ It is associated with high cardiovascular and cerebrovascular morbidity and mortality^2^ and is more prevalent among older adults.^3^ Critical limb ischemia (CLI) represents the most severe manifestation of PAD.

The UK's aging population is expected to grow significantly over the next 50 years, with an additional 8.6 million people aged 65 years and older, including the rapidly growing 85+ age group.^4^ This demographic shift increases the prevalence of PAD and the burden on vascular teams, who must manage frail, multimorbid patients.

In our region, the prevalence of PAD exceeds the national average.^5^ Additionally, the proportion of individuals aged over 60 years is projected to rise significantly by 2027. This situation poses substantial challenges for our local vascular service, as a major regional centre serving 1.5 million people, in providing safe and effective care while optimising the utilisation of limited inpatient beds.

The benefits of Care of the Elderly (CoE) input are well recognised.^3,6,7^ Frailty is widely acknowledged as a predictor of poor postoperative outcomes and prolonged length of stay (LoS),^8,9^ which was further emphasised during the COVID-19 pandemic. Recent guidelines from the General Medical Council (GMC), National Institute for Health and Care Excellence (NICE), Royal College of Surgeons (RCS), Geriatric Intervention and Support Team (GIST) and the European Society for Vascular Surgery (ESVS) emphasise the importance of preoperative optimisation and multidisciplinary collaboration for older surgical patients. NICE^10^ has recommended preoperative optimisation clinics for older patients, further reinforcing the importance of supporting elderly surgical patients. The Getting It Right First Time (GIRFT) review of vascular services also advocates for CoE collaboration with surgical specialties.

The primary aim was to reduce complications, enhance recovery and consequently reduce LoS by integrating regular input from the Proactive Care of Older People Undergoing Surgery (POPS) team, a CoE medicine branch dedicated to surgical patients on the vascular ward.

## Methods

Prospective data collection focused on the impact of CoE input on predefined parameters, with LoS as the primary outcome over a 12-month period.

The data collected included baseline demographics, comorbidities, frailty scores, LoS and referrals to medical specialties. Frailty was assessed using the Rockwood frailty score, completed by the vascular team during admission. This approach allowed the CoE consultant to see more patients and necessitated training sessions for the vascular team, particularly the Vascular Nurse Specialist. Subsequently, more parameters were identified to help focus CoE input with better case selection.

**Figure 1:**
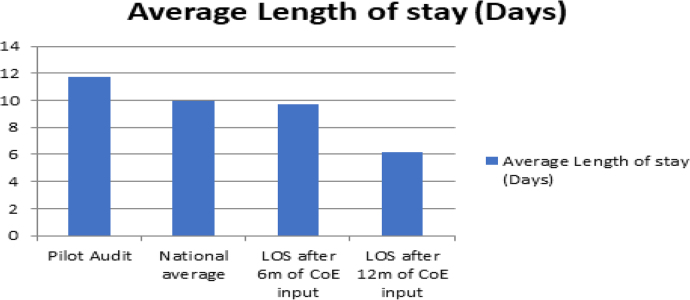
Comparing LoS before and after QIP implementation. LoS = length of stay; QIP = quality improvement project.

An earlier retrospective pilot study of 50 consecutive patients admitted to the vascular unit was conducted to demonstrate the need for CoE input in vascular patients. The pilot study revealed that the local LoS was higher than the national average (11.7 days versus 10 days; Figure 1). A total of 77% required referrals to other medical specialties, causing discharge delays, and 50% had delayed discharges after being surgically fit for discharge.

Initially, the referral age limit was set at 74+ years, consistent with the trust's standards. However, considering the pilot study's mean patient age of 70 years and the significant presence of frailty in younger adults,^11^ the referral criteria were adjusted to include patients aged 60+ years with a higher “physiological” age.

Supported by the GIRFT review's recommendations and the pilot study, funding was secured for regular CoE input on the vascular ward. Unfortunately, due to high demand and a limited number of consultants, the CoE team could provide only 1.5 clinical sessions per week. During these sessions, the consultant optimised patients preoperatively and managed postoperative complications.

Quantitative data were collected prospectively, including patient age, comorbidities, surgical procedures, LoS, complications and referrals to other specialties, allowing for comprehensive comparison with the pilot study.

## Results

Patients in both the pilot and project groups were matched for comorbidities, frailty scores and interventions (Table 1). However, the project group had a higher mean age and a greater proportion of patients aged 75+ years (77 years versus 70 years and 65% versus 40%, respectively). Despite this, we aimed to reduce LoS, acknowledging that older surgical patients face higher risks of postoperative complications and prolonged hospital stays.^9,12^

**Table 1:** Comparison of demographics and interventions

Parameter	Pilot/control group	CoE input group
Male: female	3:1	3:1
Age (mean, range)	70 (45–90)	77 (60–89)
<64 years	24%	10%
65–74 years	36%	25%
>75 years	40%	65%
Number of comorbidities	5	5
Diabetes	50%	40%
Frailty Score ≥6	42%	41%
Patients having interventions	90%	82%
Number of interventions per patient	1–4	1–3
Major amputation	38%	27%

CoE = care of the elderly

The pilot study showed that 50% of patients were discharged after being surgically fit for discharge, with a mean LoS higher than the national average (11.7 days versus 10 days). Following the quality improvement project (QIP) implementation, LoS beyond fit-for-discharge decreased from 11.7 days to 9 days within 6 months and to approximately 6 days after 12 months (Figure 1). Referrals to medical specialties significantly reduced from 77% to 40%, and the proportion of patients with new diagnoses on discharge increased from 28% to 37%.

The cohort included both elective and emergency admissions, with a high prevalence of comorbidities such as diabetes, chronic kidney disease and heart disease. Major amputations were performed in 27% of the cases, with a higher LoS associated with these patients. Common risk factors for prolonged admission and poor outcome included anaemia observed in 29% of patients, sepsis 45% of patients, renal impairment 26% and malnutrition evidenced by hypoalbuminemia 43% of patients. Patients with two or more of these factors had significantly longer LoS and higher mortality rates (Table 3).

**Figure 2:**
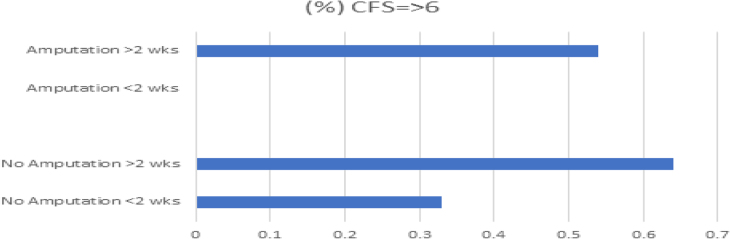
Association of major amputation and frailty scores with extended Lo. SLoS = length of stay.

Unsurprisingly, the other factors significantly associated with extended LoS were major amputation and frailty scores of six or higher. Of patients undergoing major amputation, 45% had LoS exceeding four weeks, compared with 21% for those without amputation. Although frailty scores alone were not significantly associated with increased overall LoS, a frailty score of six or higher was linked to significant delays beyond surgical fitness for discharge, particularly in major amputation cases (Table 2, Figure 2).

**Table 2:** Delayed discharge by frailty score and amputation status

Group	No. of patients	Frailty score ≥6	Delayed discharge >2 weeks
Nonamputation, <2 weeks	75	33%	0.33
Nonamputation, >2 weeks	14	64%	0.64
Amputation, <2 weeks	14	0%	0.00
Amputation, >2 weeks	24	54%	0.54

Although some trends did not reach statistical significance due to small sample size, key observations included no difference in mortality between amputation and nonamputation groups, and higher mortality in nonamputation patients with delayed discharge due to severe frailty, sepsis and renal impairment (Table 3).

**Table 3:** Comorbidities and outcomes

Parameter	Nonamputation (<2 weeks)	Nonamputation (delayed discharge>2 weeks)	Amputation (<2 weeks)	Amputation (delayed discharge>2 weeks)
Number of comorbidities	5	4.6	4.6	5.2
Frailty score ≥6	33%	64%	0%	54%
CRP >50	36%	50%	14%	45%
Albumin <35	32%	57%	8%	43%
Creatinine >128	17%	36%	0%	26%
Haemoglobin <10	21%	21%	5%	29%
30-day mortality	1.3%	43%	0%	9%

CRP = C-reactive protein

## Discussion

Myriad literature suggests that CoE input reduces postoperative complications and LoS.^13–15^ However, it is not specific to vascular patients, who represent a different group with multiple significant comorbidities and loss of physiological reserve. There is also relatively little literature looking specifically at frailty in vascular patients.

Recent systematic reviews and meta-analyses have reinforced the positive impact of comprehensive geriatric care models on postoperative outcomes, including for vascular patients. Saripella *et al* highlighted the effectiveness of interventions involving Comprehensive Geriatric Assessment (CGA) in significantly reducing postoperative complications and LoS in elderly surgical patients, including those undergoing vascular procedures.^16^

In an aging population where surgery comes with increased morbidity and mortality, it is imperative that vascular units respond to the challenges of performing procedures on this high-risk cohort of patients. In response to local pressures, we aimed to reduce LoS for patients admitted with CLI and thus improve patient outcomes and reduce bed pressures by creating collaboration with the trust's CoE team, facilitating links in an multidisciplinary team setting and developing practice capabilities required of all advanced clinical practices.^17^

Having a collaborative relationship with the CoE consultant reduced LoS from 11.7 days to 9 days in the first six months. This was followed by a further reduction to 6.2 days in the following six months. Considering that the CoE consultant could only provide 1.5 clinical sessions per week (equating to approximately 4.5 hours), the reduction in LoS was much more than we expected or planned. This intervention has improved the quality of care for vascular patients, as many common postoperative complications such as delirium and malnutrition were avoided or reduced. Eeles *et al* concluded that delirium is linked to higher rates of mortality^18^; therefore, this is highly significant. It also reduced the number of bed days, making it cost-effective, which further engaged key stakeholders.

The British Geriatric Society has highlighted a concerning gap in the number of geriatricians.^19^ We demonstrated that even a reduced amount of input from CoE can be effective in well-selected vascular patients, and this is where a robust referral pathway is so important.

The usual challenge of introducing CoE input is the cost of consultant-level involvement in patient care. However, we have demonstrated that such input reduces the number of bed days significantly, with enough cost savings to justify even more involvement in this complex, highly demanding group of patients.

We also identified a few factors that facilitate “selective” input from the CoE team that are now incorporated into our daily practice. Frailty score above six and major amputation cause significant delay in discharge. Sepsis, renal impairment, malnourishment and anaemia are associated with a higher chance of ending up with major amputation and higher 30-day mortality. Early attention to these factors and early intervention are more likely to reduce morbidity, mortality and delayed discharges in this high-risk group.

In addition to the increasingly important CoE physician input in surgical settings, it is also argued that collaboration with surgical colleagues will also improve geriatric training gaps. Throughout the study period, the CoE consultant and her team of doctors in training have been included in the vascular team's regular teaching masterclasses and journal clubs, and they, in turn, have also provided teaching to the vascular team. This has shown excellent team collaboration and demonstrated a nice teaching platform across specialties, which would benefit doctors’ education on both sides.^20^

Another significant positive balancing measure was the reduction in referrals to medical specialties in the trust. In the pilot group, 77% of patients needed referrals to medical specialties; this was reduced to 40% in the group reviewed by the CoE team. This reflects the fact that most medical complications can be reduced with CoE input as there is more emphasis on prevention or earlier intervention. Although it is noted that specialist input is still sometimes necessary, it is more specific to a defined subspecialty. This could have also impacted the reduced LoS, as patients would often have stayed in the hospital waiting for these subspecialties to review them. It has also impacted positively on these other medical specialties as it has reduced the demand for their already overstretched services.

The regular POPS team input improved how the vascular team assesses and quantifies frailty. Clinical frailty scale (CFS) tools provide a more objective assessment. Hall *et al* found that using a frailty assessment tool can improve postoperative outcomes, and reduce morbidity and mortality.^12^ This is because they highlight those patients at most risk and in most need of preoperative intervention/input. This concept was highly emphasised during the COVID-19 pandemic and is becoming a routine part of patient assessment nowadays.

Before the implementation of regular CoE input, there was no formal frailty assessment in place. The team often relied on the “eyeball” or “end of the bed” test, which can be accurate for experienced clinicians.^21^ However, it is argued that this way of assessing frailty is inconsistent and subjective.^22^ Following the implementation, 100% of patients now have a CFS assessment and documentation on admission.

The use of the referral pathway and teaching of how to complete the CFS is now a part of the induction for all new starters, meaning that it will be embedded in practice and become the new norm. In view of the recent NICE preoperative guidelines for older people that suggest preoperative clinics may improve outcomes, we are looking forward to more involvement of POPS team in outpatient settings.^10^

## Conclusion

A selective input from the CoE team in the care of vascular surgery patients had a significant impact on patient care and LoS. Focusing the effort of this in-demand service on the highest-risk vascular surgical patients through a defined referral pathway helped deliver the care effectively where it was needed most. It also revealed a positive balancing measure of a reduction in referrals to medical specialties, meaning less pressure on those already stretched services.

Given the national shortage of CoE consultants and mounting pressures on all services, selective input with multidisciplinary collaborative work has had a significant positive impact on both medical and surgical teams for the benefit of the patients and has shown cost-effectiveness; a model that can be of interest to other areas that could adopt a similar approach.
